# Study of the effectiveness of a presoaking process for reducing the additives migration from babies toys

**DOI:** 10.3906/kim-2105-40

**Published:** 2021-07-14

**Authors:** Mohand Ouidir BOUSSOUM, Aicha KENTAOUI, Beatrice GEORGE, Stephane DUMARCAY, Ahmed BOUCHERIT, Hassiba LARIBI-HABCHI

**Affiliations:** 1 Laboratory of Plant Physiology Applied to Soil-less crops, Ibn Khaldoun University, Tiaret, Algeria; 2 Wood Material Studies and Research Laboratory WMSRL, France; 3 Laboratory of Chemical Engineering, University Saad Dahlab of Blida 1, Blida, Algeria; 4 Laboratory of Functional Analysis of Chemical Process, University Saad Dahlab of Blida 1, Blida, Algeria; 5 Laboratory of Hydrogen Energy Application, University Saad Dahlab of Blida 1, Blida, Algeria

**Keywords:** PVC, migration, dop, ftir, gc-ms

## Abstract

This research aims to study a process of steeping in the n-heptane, used for reducing the migration of additives contained initially in toys for babies plasticized with di-octylphtalate (DOP) based on poly (vinyl chloride) (PVC) and stabilized with epoxidized sunflower oil (ESO). Two formulations were carried out at different levels of DOP plasticizers (15% and 45%). The migration tests were conducted in the synthetic saliva in the absence and in the presence of α-amylase with or without agitation at 37° C for 1, 3, and 6 h. The migration phenomenon was studied on the basis of preliminary studies based on the mass variation of the two formulations and where the physico-chemical technical analysis: Fourier-transform infrared spectroscopy (FTIR) and gas chromatography coupled with gas chromatography-mass spectrometry (GC-MS) were performed. This work shows that the presoak method can be used successfully to reduce the migration phenomenon of the additives and to decrease the interactions between the PVC samples and the saliva stimulant. This treatment has allowed a notable decrease of the overall migration of all the additives from saliva. It is noted that the high pH value (7.17) was obtained with the F45% formulation under agitation and in the presence of α-amylase, a mass loss of the order of 0.9004 and a minimum DOP concentration of 0.024 ppm. The analysis by GC-MS provided the DOP chromatograms of the control and the specimens, which have undergone migration tests and treatments. In addition, the amount of DOP, migrated in the case of the F15% and F45%, controls the formulations and was greater than those of the presoaked formulations, which have indicated the efficiency of the applied process. This study shows that migration has taken place, and that the soaking treatment has reduced the migration of all the additives present in the PVC samples.

## 1. Introduction

Poly (vinyl chloride) (PVC) is a polymer widely used in the plastic industry and in different sectors such as food, pharmaceutical, construction and children’s toys due to its excellent properties. However, poor thermal stability is one of its main drawbacks. It undergoes serious degradation through the removal of the hydrochloric acid at relatively low temperatures [1, 2]. Poly (vinyl chloride) (PVC) has continued to be a research topic in polymer science since its discovery in the early 19th century. Its internal structural defects, which stem from its direct manufacture (via free radical polymerization), heighten its peculiarities, including its thermal instability [3]. It has a number of qualities, such as lightness, strength and resistance, impermeability to gases and compatibility with many substances, and ease of maintenance. These properties are at the origin of the variety of its applications [4]. At all times, the play has been a powerful lever for learning for children at several levels. It is through play that the child can be able to perfect his motor, psychomotor, language, cognitive, and social-emotional skills [5].

Phthalate esters are widely used as industrial plasticizers. In particular, di-octylphthalate (DOP) is used in the manufacture of the PVC and other plastics to achieve the desired softness, flexibility, and stability for specific applications [6–11]. The low thermal stability of PVC requires the incorporation of thermal stabilizers during processing. Stabilizers based on Ca and Zn stearates are approved for food use, and their stabilizing action on PVC consists of preventing or limiting the departure of HCl in the chain [12, 13].

The release of phthalates in these various media is possible due to the weak covalent bond of these compounds to the polymers [14]. The main effects of phthalates that have been reported in experimental studies with different animal species are testicular atrophy, liver damage, decreased fertility, decreased fetal weight, increased kidney mass, antiandrogenic activity as well as teratogenic effects [14].

The use of certain phthalates in toys could cause health risks for children and tends to escape from the polymer matrix either by evaporation, by liquid extraction or by diffusion into another solid [15]. The exposure is, therefore, greater and chronic for children compared to adults because they are in an important phase of their growth [16]. In recent years, special attention has been paid to plasticized PVC toys. It has been pointed out by many researchers [14, 17] that babies and toddlers suck and chew on their toys so that the phthalates dissolve and escape into the baby’s saliva, putting them at risk. What are ignored, however, are the precise amounts of migrating plasticizers and the precise duration of children’s exposure to plasticizers as well as the precise nature of the damage to children exposed to flexible PVC.

In recent years, special attention has been paid to toys made from plasticized PVC which contain significant amounts of phthalates and which can cause kidney and liver damage and cancer.

Our work aims to study the migration and the presoaking process effect of plasticizer (DOP), initially contained in PVC stabilized with epoxidized sunflower oil (ESO), in synthetic saliva in order to study the factors favoring this process and to quantify the migrant substance. Two formulations at different levels of DOP plasticizers (15% and 45%) were carried out. The choice of these two levels of DOP plasticizers was considered in order to study the rigidity or flexibility of the toys on the rate of mass change of the PVC specimens in contact with saliva.

Migration tests on synthetic saliva in the presence and absence of α-amylase enzyme were conducted with and without agitation at 37° C and at different contact times (1, 3, and 6 h). The migration phenomenon was based on the rate of mass variation of the PVC specimens in contact with the synthetic saliva. The physico-chemical analytical techniques, Fourier-transform infrared spectroscopy (FTIR) and gas chromatography coupled with gas chromatography-mass spectrometry (GC-MS), were performed.

## 2. Experimental

### 2.1. Materials

A PVC suspension (Petvinil S39/71) produced by SO.G.I.S.SPA (Italy) was used. Zn and Ca stearates from Aldrich (France), di-octylphtalate (DOP) (Mw = 390.6 g/mol) from SGP (Tunisia) and stearic acid (stearine JOS) from SO.G.I.S.SPA (Italy) were used as received. Heptane (99%) from Ph Eur-reagent (Germany), tetrahydrofuran (THF 99.5%) from Ph Eur-reagent (Germany), methanol ( 99%) from Safety Card (France), and chloroform (99.8 %) from Safety Card (France). The epoxidized sunflower oil (ESO) was especially prepared as described previously [18]. The level of oxirane oxygen was 6.4%. The synthetic saliva contained 147.0 mg of CaCl_2_ (96%), 753.1 mg of K_2_HPO_4_ (99%), 525.2 mg of K_2_CO_3_, 327.3 mg of NaCl (99%), 745.3 mg of KCl (99%) and 3000 mg of α-amylase according to the kind of simulant, and finally the pH value was adjusted to 6.8 with concentrated HCl (37%) [19].

### 2.2. Preparation of PVC films

Formulations contained 1 wt.% of Zn stearate and 1 wt.% of Ca stearate, 5 wt.% of ESO, 1 wt.% of stearic acid, and two concentrations of 15% and 45 wt.% plasticizers were prepared. PVC and additives were mixed in a two- roll mill at 140° C for 20 min and melt compressed at 170° C for 5 min under a pressure of 300 kN/m^2^ in order to obtain the desired thickness (2 ± 0.1 mm) [17].

### 2.3. Presoaking procedure

The surface extraction method involves soaking the material in a plasticizer solvent and then drying it. Due to this treatment, the distribution of the plasticizer becomes nonuniform in the material. The result is a rigid surface that will slow down the exchange phenomena between the material and the media in contact.

### 2.4. Procedure for migration tests

Migration tests were carried out over a period of 1, 3 and 6 h with or without presoaking process. For this purpose, each specimen was immersed in 100 mL of saliva simulant medium in the presence or absence of the α-amylase enzyme at a temperature of 37 °C with and without agitation [9]. The surface area of the PVC pieces intended to undergo the migration tests was 10 cm^2^. The aim of the agitation was to simulate the action of the chewing; 20 glass beads of 4 mm and 30 mm diameters were introduced into a migrating cell, which was a 150 mL bottle of hermetically sealed capacity immersed in a water bath. After each test, the specimen was wiped and weighed using an accurate analytical balance with a precision of 0.1 mg.

The aim of the agitation was to simulate the action of the chewing, 20 glass beads of 4 mm and 30 mm diameters were introduced into a migrating cell, which was a 150 mL bottle of hermetically sealed capacity immersed in a water bath.

The rate of mass variation was determined following the relation [20]:


(1) $$\tau (\%)=[({\text{m}}_{1}-{\text{m}}_{0})/{\text{m}}_{0}]\times 100$$

where: m_0_ = initial mass before immersion, m_1_ = mass of the sample at the time t.

### 2.5. FTIR spectroscopy analysis

The PVC circular samples were dissolved in tetrahydrofuran (THF). After evaporation of the solvent, a polymeric film was recovered and analyzed with a Vector 22 (Buker) FTIR. The resolution was 2 cm^−1^.

### 2.6. GC-MS analysis

GC-MS analysis was performed on a Perkin–Elmer GC connected with a MS detector. A 30 m capillary column apolar stationary phase DB5-5MS (5% diphenyl ether, *≥* 99%, 95% dimethyl polysiloxane E(900)) of 0.25 μm diameter was used. The analysis was carried out using electron impact mode and an ionization potential of 70 eV. The carrier gas was helium with a flow of 2 mL/min.

The preparation of the samples for GC-MS analysis was carried out according to the protocol described by Wang and Storm [21], and Fantoni and Simoneau [22]. About 0.1 g of the PVC sample was dissolved in 5 mL of tetrahydrofuran (THF) and precipitated by adding a volume of methanol equal to 2.5 the volume of THF, thus, separating the filtrate from the PVC and drying it at 80° C for 30 min. The residue was dissolved in 1 mL chloroform.

The analysis was conducted under the following conditions: 90 °C held for 3 min, heated up to 250° C at a rate of 6° C/min and held for 13 min. Molecular mass in the range 50–450 amu was scanned. The identification of the different peaks was deduced by searching in the MS library (NIST) and further confirmed by running the known chemical for DOP. The quantification was performed using m/Z of 149. Calibration curve for DOP was prepared in chloroform at concentrations that covered the concentration range found in the polymer extracts. The resulting line was linear with a correlation coefficient of 0.9977. Three analytical replicates were analyzed for each concentration.

## 3. Results and discussion

### 3.1. Study of the evolution of the Ph value variation

**Figure 1:**
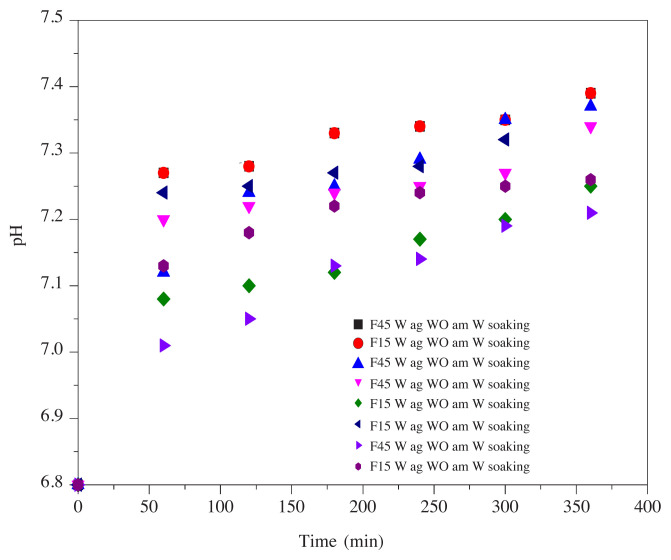
pH value variation with contact time in synthetic saliva at 37 °C.

The results in Figure 1 show that the pH value variation of the saliva specimens undergoing the migration tests is higher than that of the saliva samples subjected to the presoak treatment, demonstrating the effectiveness of the migration reduction of all the additives [1].

### 3.2. Study of global migration based on mass variation

**Figure 2:**
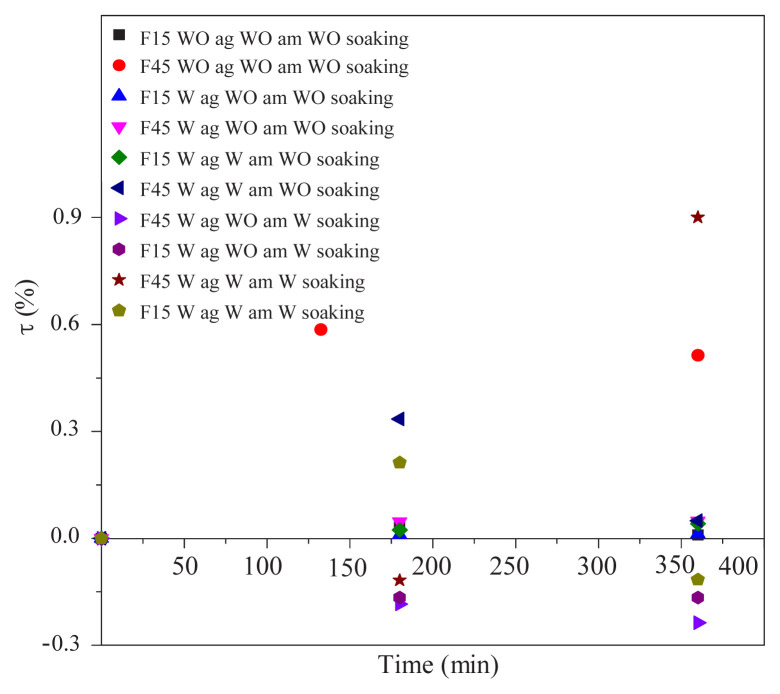
Evolution of the rate of mass variation (τ) with contact time.

The results illustrated in Figure 2 show that the rate of mass variation for the F45% formulation is higher than that of the F15% formulation, which is linked to the initial DOP content.

The curves are increasing in shape due to the penetration of the saliva into the PVC test specimens, then decreasing patterns are observed due to the migration of certain additives to the synthetic saliva. On the other hand, a growth in the curve obtained in the presence of the α-amylase enzyme indicates the penetration of saliva into the PVC test pieces.

The amount of compounds absorbed is directly proportional to the concentration of the additives. At higher concentrations, the migrating amounts can even modify the polymer matrix [23]. It can be noted that the increase in the number of the additives in the formulation decreases the transfer resistance at the liquid-solid interface as well as the internal diffusion resistance of the additives in the polymer matrix [24]. These results indicate also that the rates of mass variation of the F15% and F45% formulations conducted under agitation are higher than those conducted in the absence of agitation. These results are consistent with those obtained by Boussoum et al. [17]. The curves of F15% and F45% formulations without agitation have two phases an increasing phase, which means a gain in mass due to penetration of the saliva and the other decreasing, which means a loss of mass of the specimens which indicates that the migration has taken place. On the other hand, the curves of the two formulations in the presence of α-amylase have increasing curve, which indicates the phenomenon of penetration. The migratory phenomenon was influenced by agitation [15], which tolerates a good additive solubility and the renewal of the liquid in contact with the material, causing the rearrangement of the molecules at the surface of test pieces. This will increase the free surface and the volume of the pores, which allows the passage of saliva into the interior of the test pieces.

These results indicated that the rate of mass variation for the two F15% and F45% formulations with α-amylase is lower than that in the absence of the α-amylase enzyme. The sorption of the molecules in a polymeric film depends strongly on their polarity and their hydrophobicity, which are both linked [25]. The contact simulant (food) can penetrate into the material according to their reciprocal affinity [15]. Finelly, these results showed that presoaking process has an effect on the decrease in the rate of mass variation of the simulant considered. Indeed, when the PVC samples were presoaked in the n-heptane, this treatment has modified the concentration profiles of the additives in the PVC test pieces, which has influenced on the migrating quantity [15]. The values of the overall migration shown in table 1 are below the recommended limit of (10 mg/dm^2^) [26] for both types of tests. In addition, the smallest global migrations values are obtained in the case of presoaking tests.

### 3.3. FTIR spectrometry analysis

**Figure 3:**
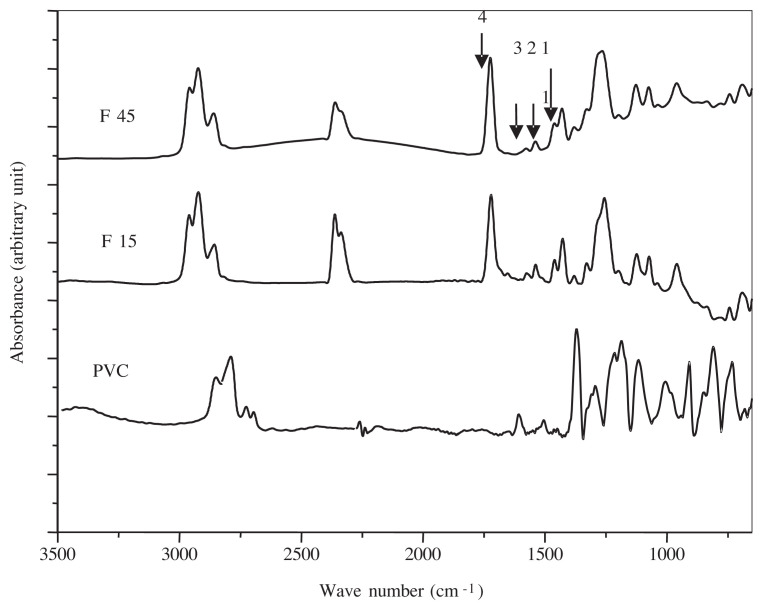
FTIR spectra of PVC alone, PVC containing 15% and 45% DOP.

The direct analysis of the saliva stimulant spectra did not show the migration of additives because of the overlapping of the characteristic bands and the very low amounts of additives migration. However, the investigation of the PVC films spectra confirmed that some migration of the DOP and other additives (ESO, Zn and Ca stearates) has occurred. The comparison of the spectrum of PVC alone and the spectrum of PVC with the additives (Figure 3) allowed the identification of some characteristic bands, which are related to the additives present in the formulation. For that purpose, the following absorbance ratios were calculated:

1. A1457/A1426: ESO, Ca and Zn stearates complexes migration.
2. A1541/A1426: Ca and Zn stearates complexes migration.
3. A1719/A1426: Ca and Zn stearates complexes migration.
4. A1731/A1426: ESO and DOP migration.

**Figure 4:**
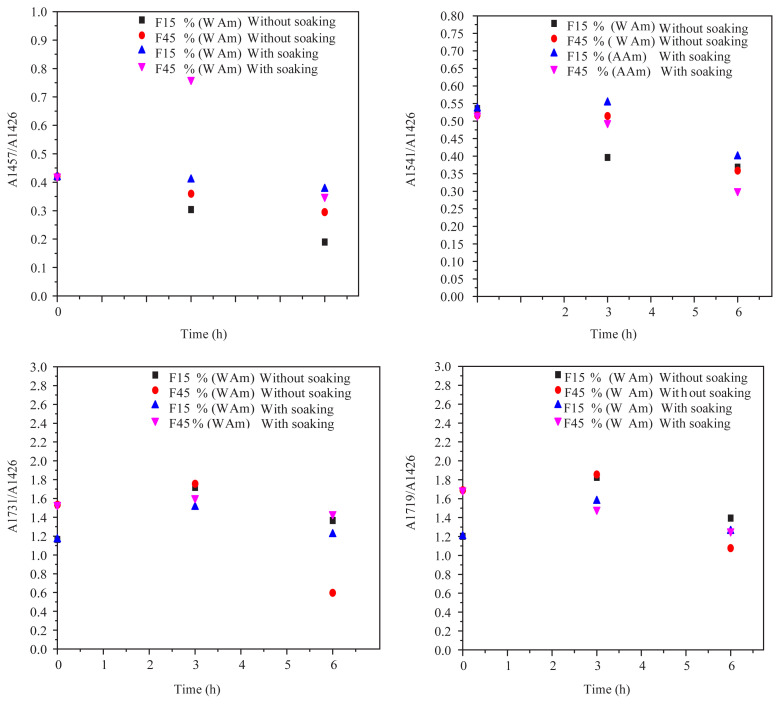
Variation of absorbance ratios as a function of contact time of saliva simulant.

The band at 1426 cm^−1^ was due to the vibration of CH_2_ of PVC [23] and was taken as a reference band. The variations of these four absorbance ratios as a function of the contact time for synthetic saliva with the two F15% and F45% formulations are illustrated in Figure 4. An initial increase is first observed, indicating the penetration of simulator medium into the PVC specimens. It is followed by a decrease of all the curves with contact time. This can be directly related to a phenomenon of migration of ESO, DOP, Zn, and Ca stearates complexes and stearine in synthetic saliva. It seems that the penetration of simulator medium into the PVC specimens favored the mobility of the additives and their migration. On the other hand, the highest absorbance ratios were obtained for the PVC samples soaked in the n-heptane. This feature indicates that residual concentrations of these additives are higher in comparison with samples without soaking. Then, it is obvious that the highest migration occurred in the case of the samples without soaking.

### 3.4. GC-MS analysis

**Figure 5:**
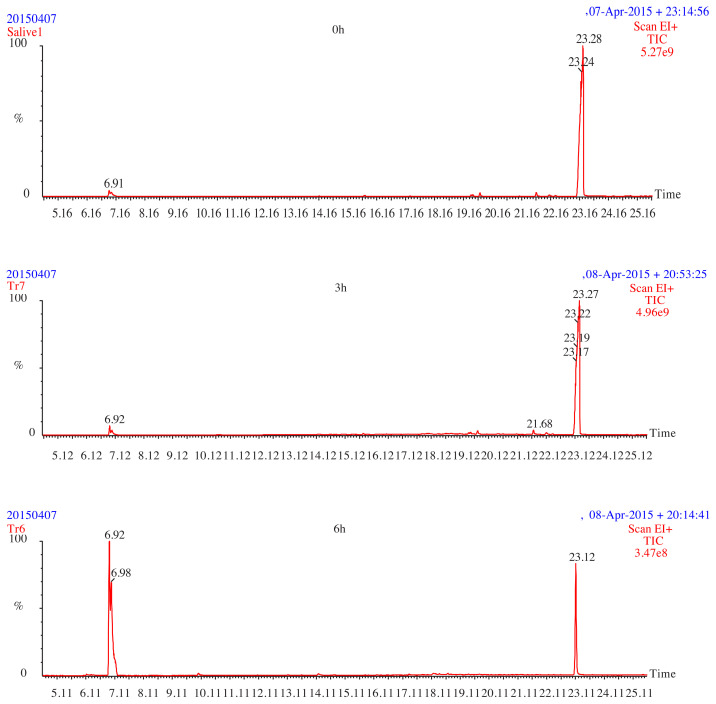
Chromatograms of the 45% DOP test specimens presoaked in the n-heptane and brought into contact with the saliva with agitation and without α-amylase.

The determination of the migrated DOP in each specimen was achieved by GC-MS (Figure 5).

The corresponding values show clearly that the soaking treatment has decreased considerably the migrations of DOP (Table 2).

According to this table, it appears that

- The amount of the migrated DOP in the case of specimens that have been tested for saliva migration in the presence of the enzyme is greater than that determined in saliva in the absence of the enzyme, which means that the nature of the medium simulator plays a very important role in the transfer of DOP, since each simulator behaves differently by its physico-chemical properties (degree of affinity presented towards the DOP).
- The amount of the migrated DOP in the case of the F45% formulation is greater than that of the F15% formulation, which shows the influence of the initial DOP content on the migration phenomenon.
- The amount of the migrated DOP in the case of the two formulations studied under the effect of agitation is greater than that in the absence of agitation which shows the influence of agitation on the migratory phenomenon.
- Presoaking process decreased DOP migration.

All these together confirm the results obtained during the estimation of the migration by the study of the rate of the mass variation, the evaluation of the pH value and the humidity rate, and especally by the FTIR and UV-visible spectroscopy analysis.

## 4. Conclusion

The present work has shown that the presoaking methodology can be used successfully in order to decrease the migratory phenomenon of the additives (ESO, DOP, Zn, Ca stearates complexes and stearine). The monitoring of the evolution of the pH value of the two simulators media demonstrates the existence of interactions between the PVC test pieces and the saliva. The rate of mass change in saliva without α-amylase was greater than that with α-amylase. The FTIR spectra has allowed us to highlight the migration of ESO, DOP, Ca and Zn stearate complexes due to a semiquantitative estimation based on the evolution of the absorbance ratios as a function of contact time. The GC-MS analysis has permitted us to obtain the DOP chromatograms of the control and the specimens which have undergone migration tests and those which have undergone treatments. In addition, the amount of DOP, migrated in the case of the two F15% and F45% formulations, controls the formulations and was greater than those of the presoaked formulations, which have indicated the efficiency of the applied process.

Belhaneche-Bensemra et al. [27] found that the rate of migration of plasticizer in sunflower oil at 45 °C in contact with soft PVC films increases with the concentration of plasticizer by a factor of 9% for DOP contents in films of 40, 50, 60%. On the other hand, our investigation [15] allowed us to record a rate of 10.25% without treatment to 1.75% with treatment in the case of F45 formulation, which shows the effectiveness of the presoaking process.

## Figures and Tables

**Table 1:** Estimation of the overall migration (mg/dm^2^) in synthetic saliva at 37° C.

Formulations	Without presoaking (mg/dm^2^)			With presoaking(mg/dm^2^)	
	Without agitation, without α-amylase	With agitation, without α-amylase	Withagitation, with α-amylase	With agitation, without α-amylase	With agitation, with α-amylase
F15	0.466	0.650	0.366	0.450	0.300
F45	1.700	1.833	1.533	0.550	0.400

**Table 2:** Concentration of DOP in the extract of the control formulation and in the formulations that have been subjected to the migration tests.

Formulations		F45%					F15%				
[DOP] (ppm)	Time (Hours)	Without presoaking			With presoaking		Without presoaking			With presoaking	
		without α-amylase		Wam & W ag	W ag, WO am	W am & W ag	without α-amylase		Wam & Wag	WOam & Wag	W am & Wag
		WO ag	W ag				WO ag	W ag			
0		0.04	0.04	0.04	0.04	0.04	0.02	0.02	0.02	0.02	0.02
1		0.052	0.044	------	------	------	0.019	0.018	---	----	-------
3		0.048	0.024	0.051	0.025	0.039	0.15	0.15	---	0.0139	0.019
6		0.0041	0.0007	0.038	0.007	0.024	0.014	0.0136	0.017	0.0137	0.0719

WO ag:Without agitation; W ag: With agitation; WO am: without α-amylase; W am: with α-amylase.
